# Simultaneous production of two kinds of sounds in relation with sonic mechanism in the boxfish *Ostracion meleagris* and *O. cubicus*

**DOI:** 10.1038/s41598-019-41198-x

**Published:** 2019-03-21

**Authors:** Eric Parmentier, Laura Solagna, Frédéric Bertucci, Michael L. Fine, Masanori Nakae, Philippe Compère, Sarah Smeets, Xavier Raick, David Lecchini

**Affiliations:** 1 Université de Liège, Laboratoire de Morphologie Fonctionnelle et Evolutive, AFFISH-RC, Institut de Chimie - B6C, Sart Tilman, Liège, 4000 Belgium; 2 PSL Research University: EPHE-UPVD-CNRS, USR3278 CRIOBE, BP 1013, 98729 Papetoai, Moorea French Polynesia; 3 Department of Biology, Virginia Commonwealth University, Richmond, Virginia 23284-2012 USA; 4 Department of Zoology, National Museum of Nature and Science, 4-1-1 Amakubo, Tsukuba, Ibaraki 305-0005 Japan; 5 Laboratoire d’Excellence «CORAIL», BP 1013, 98729 Papetoai, Moorea French Polynesia

## Abstract

In fishes, sonic abilities for communication purpose usually involve a single mechanism. We describe here the sonic mechanism and sounds in two species of boxfish, the spotted trunkfish *Ostracion meleagris* and the yellow boxfish *Ostracion cubicus*. The sonic mechanism utilizes a T-shaped swimbladder with a swimbladder fenestra and two separate sonic muscle pairs. Extrinsic vertical muscles attach to the vertebral column and the swimbladder. Perpendicularly and below these muscles, longitudinal intrinsic muscles cover the swimbladder fenestra. Sounds are exceptional since they are made of two distinct types produced in a sequence. In both species, humming sounds consist of long series (up to 45 s) of hundreds of regular low-amplitude pulses. Hums are often interspersed with irregular click sounds with an amplitude that is ten times greater in *O. meleagris* and forty times greater in *O. cubicus*. There is no relationship between fish size and many acoustic characteristics because muscle contraction rate dictates the fundamental frequency. We suggest that hums and clicks are produced by either separate muscles or by a combination of the two. The mechanism complexity supports an investment of boxfish in this communication channel and underline sounds as having important functions in their way of life.

## Introduction

Recent years have been marked by progress in the field of aquatic communication, notably increasing work on diversity of the acoustic communication at sea^1,2^ and the impact of anthropogenic sounds on fish populations^3–5^. Fish acoustic behavior is a prominent feature of coral-reef environments, and a wide variety of fishes produces specific courtship, spawning and agonistic sounds^6,7^. Coral reefs house approximately 50 fish families and about 30% of all fish species are able to produce sounds. On this basis, Lobel and colleagues nicely propose this biotope could be called “choral reef”^7,8^.

Boxfishes of the family Ostraciidae (Tetraodontiformes) occur in shallow tropical and warm seas of the world. The name stems from the body being almost completely encased in a bony shell or carapace formed of enlarged, thickened scale plates, usually hexagonal in shape, that are firmly sutured to one another^9^. The spotted trunkfish *Ostracion meleagris* Shaw, 1796 is a protogynous hermaphrodite species that is sexually dichromatic. Juveniles and females are uniformly dark brown with small white spots. The dorsal surface of males is black with white spots, but the flanks are blue with dark-edged orange-yellow spots on the side, and the belly is grey. This species is found in Indo-Pacific coral reefs, between the surface and 30 m depth^10^. Boxfishes are generally territorial with males defending groups of females. They tend to spawn after sunset^11^. The male approaches a female, and they both ascend with his snout against her back. Gamete release takes place when the pair lines up side by side, and both curl their caudal fins in opposite directions^12^. In the yellow boxfish *Ostracion cubicus* Linnaeus, 1758, males show brilliant yellow sides and a blue dorsal surface. Females are basically yellowish-brown^10,13,14^. *Ostracion cubicus* are also protogynous hermaphrodites and form harems of 2–4 females for each male. Mating behavior appears similar to *O. meleagris*, but further studies are required^15^.

Sounds have been recorded from this family. In the thornback cowfish *Lactoria fornasini*, high-pitched hums, although physically undescribed, are thought to help synchronizing spawning (Moyer 1979). In *Ostracion meleagris*, Lobel^16^ identified three different sounds. Spawning sounds have long durations (6 s) with peak energy between 215 and 270 Hz and are produced when a pair is in mating position. During competition for mates, males also produce “bump” sounds with a duration ranging from 9.9 to 10.6 ms and peak energy between 140 and 790 Hz. The third sound, the “buzz”^16^ is probably related to agonistic behavior^17^. It has peak energy between 198 and 535 Hz and durations of 130 to 209 ms.

Recent reviews have underlined the growing diversity of sound-producing mechanism in fishes^2,18–20^. Most mechanisms, resulting from evolutionary convergences, employ high-speed muscles (contraction rate between 50 and 300 Hz) that insert in part or totally on the swimbladder. In these species, the contraction rate dictates the fundamental frequency of the sounds^21,22^. The sound-producing mechanism of the Ostraciidae is not known. As in triggerfishes (Balistidae, Tetraodontiformes), the swimbladder is a voluminous elongated thick-walled dorsal sac with two anterior evaginated lateral lobes^23^. Fish and Mowbray proposed the use of sound- producing muscles related to the swimbladder and jaw teeth stridulation for the genus *Lactrophrys*, but they provide no evidence and did not describe the muscles. There is no indication for other genera of Ostraciidae such as *Ostracion*^24^. This study aims to examine for the first time the fish inner anatomy and sound features to infer the sonic mechanism in two boxfish species, *Ostracion meleagris* and *Ostracion cubicus*.

## Material and Methods

Experimental procedures followed a protocol approved by the local ethics committee of the University of Liège. *Ostracion meleagris* and *O. cubicus* are not endangered or protected species, and specimens were not caught in protected areas at Moorea Island. All procedures were approved by the ethical commission of the University of Liège (ethics case 1759).

### Biological material

A field campaign was conducted at the CRIOBE research station (www.criobe.pf) in Moorea Island (French Polynesia) between February and April 2016. Twenty-seven *Ostracion meleagris* from 3 to 9 cm in standard length (twelve males, eight females and seven juveniles) and five *Ostracion cubicus* adults (7 to 12 cm) were captured in the North Lagoon of Moorea. They were caught inside rocky crevices in the reef in water from 1 to 3.5 m depth using a gillnet (25 m long and mesh of 2.5 cm) and hand nets. Fish were stocked in two tanks (137 cm × 68 cm × 60 cm) with running seawater (28–29 °C) on a natural light cycle (12 h light:12 h dark). The tanks were equipped with aerators for oxygenation and stones and coral fragments as shelters. Fish were fed shrimp three times a week, but they also fed on algae on the stones. Barriers separated *Ostracion meleagris* in order to avoid fighting between males.

### Morphological study

Nine *O. meleagris* (five males and four females) and one male *O. cubicus* were euthanized with an overdose of MS 222 (Sigma Aldrich). Three males and two females were fixed in 7% formalin for approximately two weeks before transfer to 70% ethanol. Swimbladders were dissected and examined with a Wild M10 (Leica) binocular microscope. A second lot of *O. meleagris* (four males and four females) was dissected to sample sound-producing muscles associated with the swimbladder. Muscle samples were immediately fixed in glutaraldehyde 2.5% in 0.1 cacodylate buffer pH 7.4, post-fixed in 1% osmium tetroxide, dehydrated through a graded ethanol-propylene oxide series and embedded in epoxy resin (SPI-PON 812, SPI-CHEM). Transverse semi-thin sections (1 µm) were cut using a diamond knife on a Reichert-Jung Ultracut E ultramicrotome (Leica) and stained with toluidine blue (1%, pH 9.0) following the protocol of Richardson^25^, and photographed under a binocular microscope. Ultrathin sections (70–80 nm) were classically stained with uranyl acetate and lead citrate and viewed using transmission electron microscopy with a TEM/STEM Tecnai G2 Twin (FEI) working at 200 kV accelerating voltage.

### Sound recording

After an acclimation period of two days, fish were recorded with a hydrophone (HTI Min-96, sensitivity: −163.9 dB re 1 V μPa^−1^; flat frequency response range between 2 Hz and 30 kHz; Long Beach, MS, USA) placed in the center of the aquarium (98 × 47 × 48 cm) and connected to a Tascam DR-05 recorder (TEAC, Wiesbaden, Germany). Fish were hand held with the dorsal fin blocked in the tank approximately 3 cm from the hydrophone. Fish were held until sounds were obtained but sessions did not last more than three minutes. Different recordings of the same specimens were successively realized when necessary. Although handling has a level of artificiality, it does provoke fish to produce sounds as if they were captured by a predator^26^. This recording method was chosen because it elicits sounds from the same behavioral context, and it ensures that sounds are produced at the same distance from the hydrophone that was placed in the same part of the tank in order to avoid differences in signal loss^27–30^. Moreover, this distance also enables fish to remain within the attenuation distance^31^. With this methodology, feature variations within the sounds are due to the fish and not to environmental conditions. Ten sounds of each type were analyzed for each fish. After the recording, each fish was weighed and measured.

### Sound analysis

Sound description is based on sounds from the 27 *Ostracion meleagris* and 5 *Ostracion cubicus*. Sounds were digitized at 44.1 kHz (16-bit resolution) and analyzed with AviSoft- SAS Lab Pro 5.2 software (Avisoft Bioacoustics, Glienicke, Germany). Recordings in small tanks induce potential artifacts because of reflections and tank resonance, and an estimated minimum resonant frequencies of 2360 Hz was calculated following Akamatsu *et al*.^31^. A band-pass filter (cutting frequencies: 0.05–2 kHz) was therefore applied to all recordings. Since calls are made of trains of pulses, the following temporal acoustic variables were manually measured on oscillograms: call duration (time from the beginning to the end of the sound, ms), pulse period (time between the onset of two consecutive pulses, ms), pulse duration (time from the beginning to the end of the pulse, ms) and the number of pulses in the sound. Spectral characteristics were obtained from power spectra [Fast Fourier Transform (FFT): 512 points, Hamming window, frequency resolution 4 Hz] after converting them to 4 kHz (16-bit resolution). Dominant frequency (frequency component with greatest energy, Hz) and its associated amplitude (sound pressure level in kPa at 3 cm) were measured.

### Statistical analysis

Normality of data was assessed with the Shapiro–Wilk test. Acoustic features and biometric data (size and weight) were correlated by means of Spearman’s correlation tests. The characteristics of acoustic features in hums and click sounds within *Ostracion meleagris* (juveniles, females, males) were analyzed using a non-parametric Kruskal-Wallis test followed by a subsequent Dunn’s multiple comparison test for pairwise comparisons between categories. Due to low sample size, no intraspecific comparisons were made in *O. cubicus*. A *t*-test was used to compare acoustic features of hums and clicks between *Ostracion meleagris* and *O. cubicus*. Statistical analyses were carried out with R 3.0.2 or GraphPad (version 5, GraphPad software, Inc.).

## Results

### Morphology

**Figure 1:**
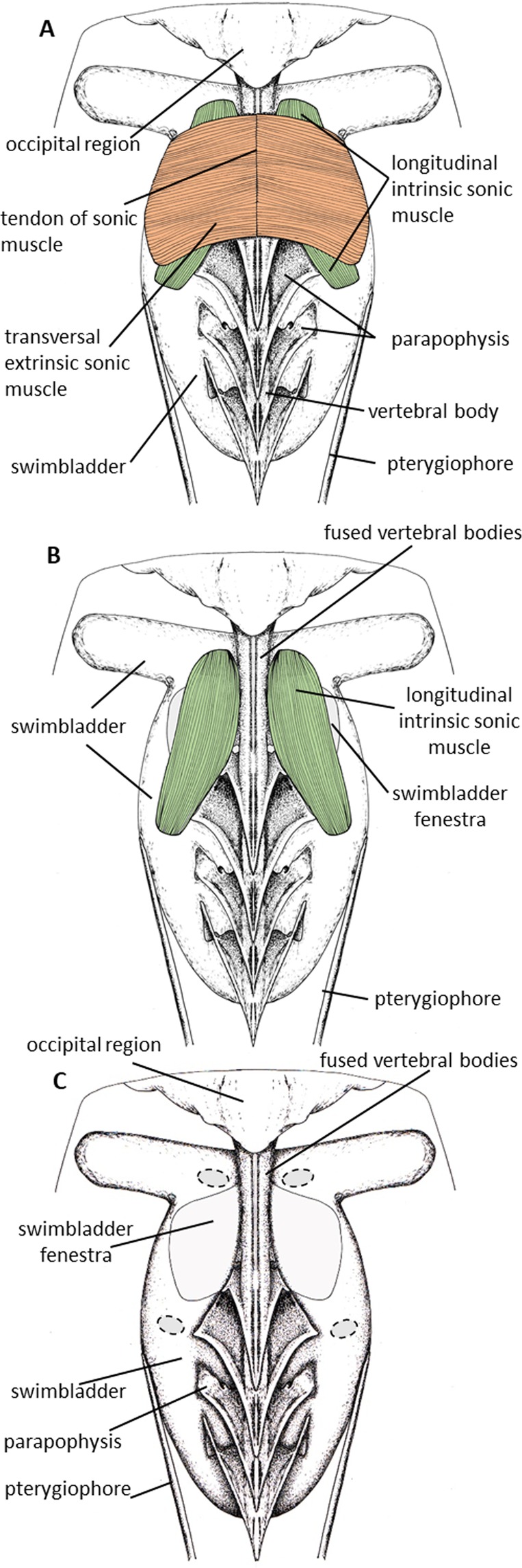
Dorsal views showing the sound producing apparatus in *Ostracion meleagris*. Muscles were progressively removed from (**A**–**C**). Drawings were made by E.P.

The swimbladder is situated under the vertebral column in an area characterized by the fusion of several abdominal vertebrae with each other and to the skull, forming an osseous complex^32^. Therefore one can discern neural spines and some spinous processes, but the individual vertebral bodies are not clearly distinguishable. The anterior vertebral column is rod like, but posterior vertebrae show enlarged parapophyses (Fig. 1). The swimbladder is shaped like a large “T”: it is composed of a voluminous thick-walled dorsal sac with two prominent lateral anterior lobes (Fig. 1). Behind the lateral lobes, the anterior part of the swimbladder has a broad dorso-lateral area that is deprived of a tunica externa, forming a swimbladder fenestra. The fenestra is partly masked dorsally by the vertebral column that divides it in two equal parts (Fig. 1C). Moreover, two elongated pterygiophores originating from the anal fin form a vice on either side of the posterior swimbladder to which they are laterally attached with connective tissue (Fig. 1C).

**Figure 2:**
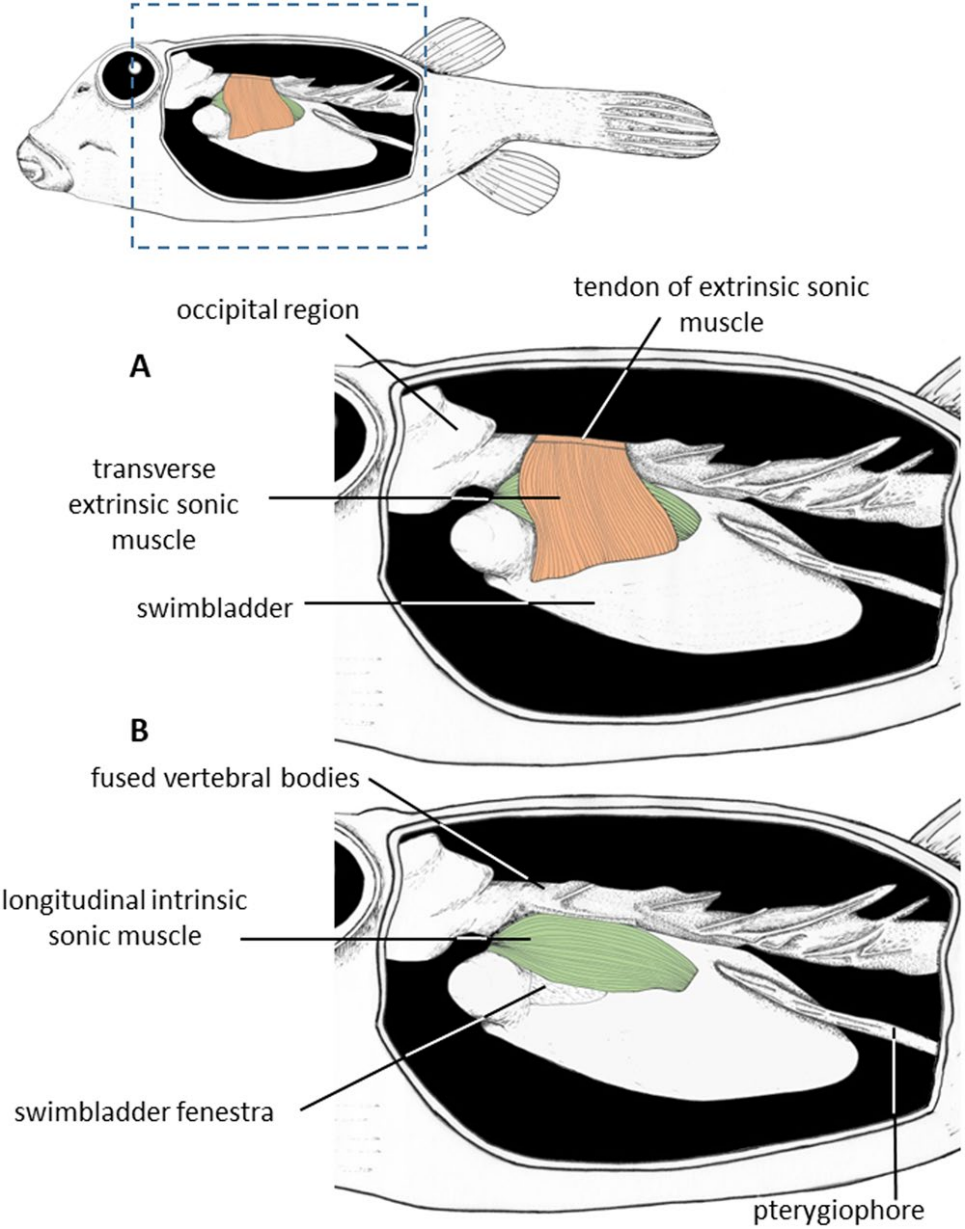
Left lateral views showing the sound producing apparatus in *Ostracion meleagris*. Transverse extrinsic sonic muscle is removed in (**B**). Drawings were made by E.P.

*Ostracion meleagris* possess two pairs of sound-producing muscles that cross at right angles above the fenestra (Figs. 1 and 2). The superficial pair is extrinsic, originating on a tendon (Figs. 1A and 2A) that attaches to the dorsal side of the vertebral column. The muscles insert on the left and right ventral edges of the swimbladder just beyond the fenestra, and the muscle fibers run laterally. The deep pair is intrinsic to the swimbladder, running between the anterior and posterior vertical edges of the swimbladder fenestra. The muscle fibers run parallel to the long axis of the fish. Therefore, the longitudinal intrinsic muscles are perpendicular to the extrinsic sonic muscles and cover the swimbladder fenestra.

**Figure 3:**
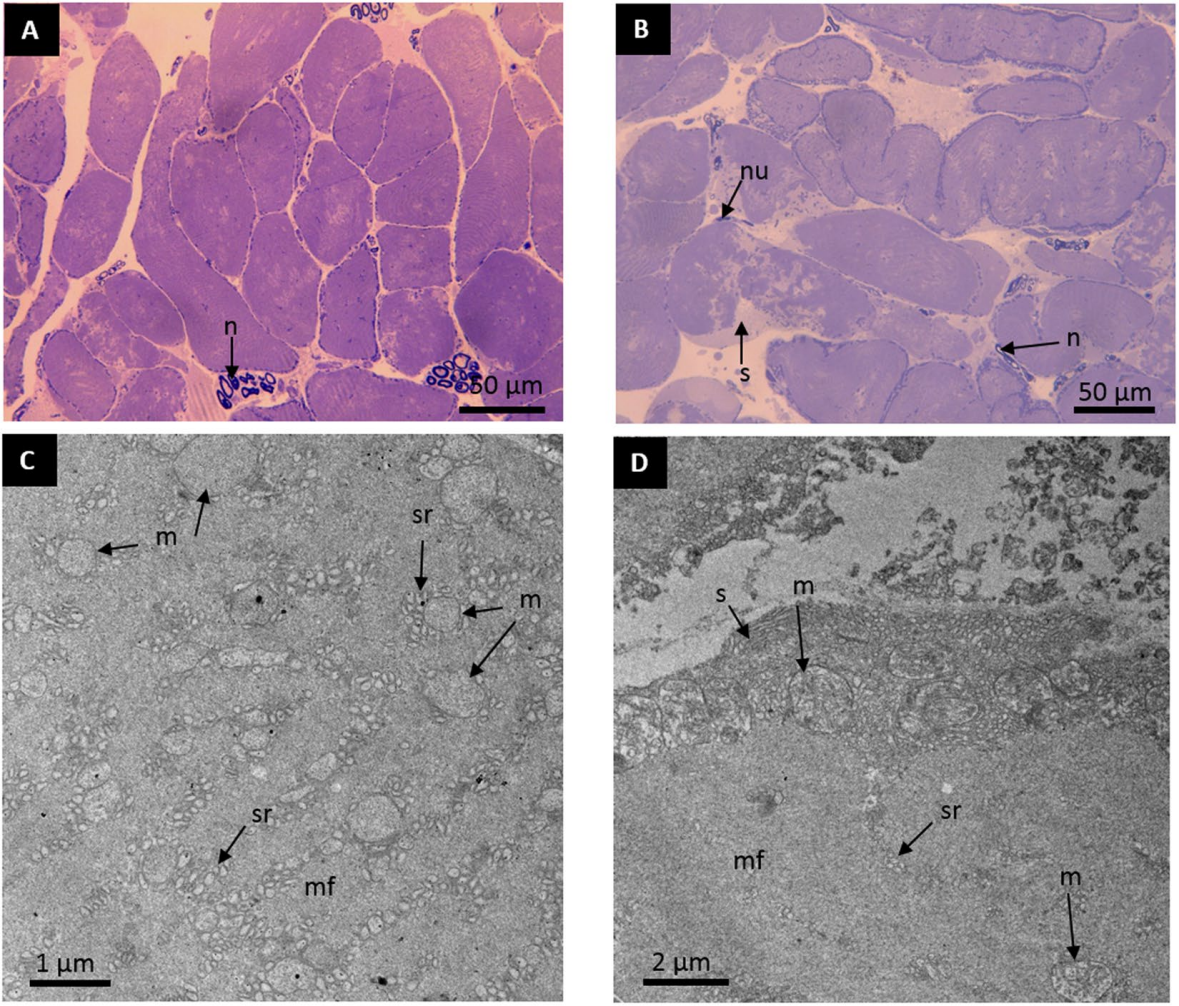
Toluidine blue-stained cross-sections of (**A**) the longitudinal intrinsic and (**B**) the transverse extrinsic sonic muscles in *Ostracion meleagris*. TEM cross-sections of (**C**) the longitudinal intrinsic and (**D**) the transverse extrinsic sonic muscles in *Ostracion meleagris* showing the central area (**C**) and the margin (**D**) of fibers. Deep blue dots around the cells (in **A** and **B**) correspond to the mitochondria; m: mitochondriae; mf, myofibril bundles; n: myelinated nerve; nu: nucleus; sr: sarcoplasmic reticulum tubules; s: sarcoplasm.

Histological sections and TEM views (Fig. 3) show many common characteristics in both muscles. They possess mitochondria of different sizes and are also characterized by a dense concentration of sarcoplasmic reticulum tubules: larger mitochondria occur mainly under the sarcolemma in the fiber periphery whereas smaller ones are distributed throughout the cell interspersed in myofibril-rich areas. In both case, they are generally close to sarcoplasmic reticulum that appear as tubule-rich areas just beneath the sarcolemma at the cell periphery or between fibril bundles. Cell morphology is however variable: some fibers show large spaces deprived of myofibrils and filled with sarcoplasmic reticulum while other cells showed myofibrils over the whole sectioned surface. Both muscles possess many nerve endings, and myelinated nerve sections are obvious on the semi-thin slices. They appeared as dark blue ring-shapes between the medium-blue fibers.

**Figure 4:**
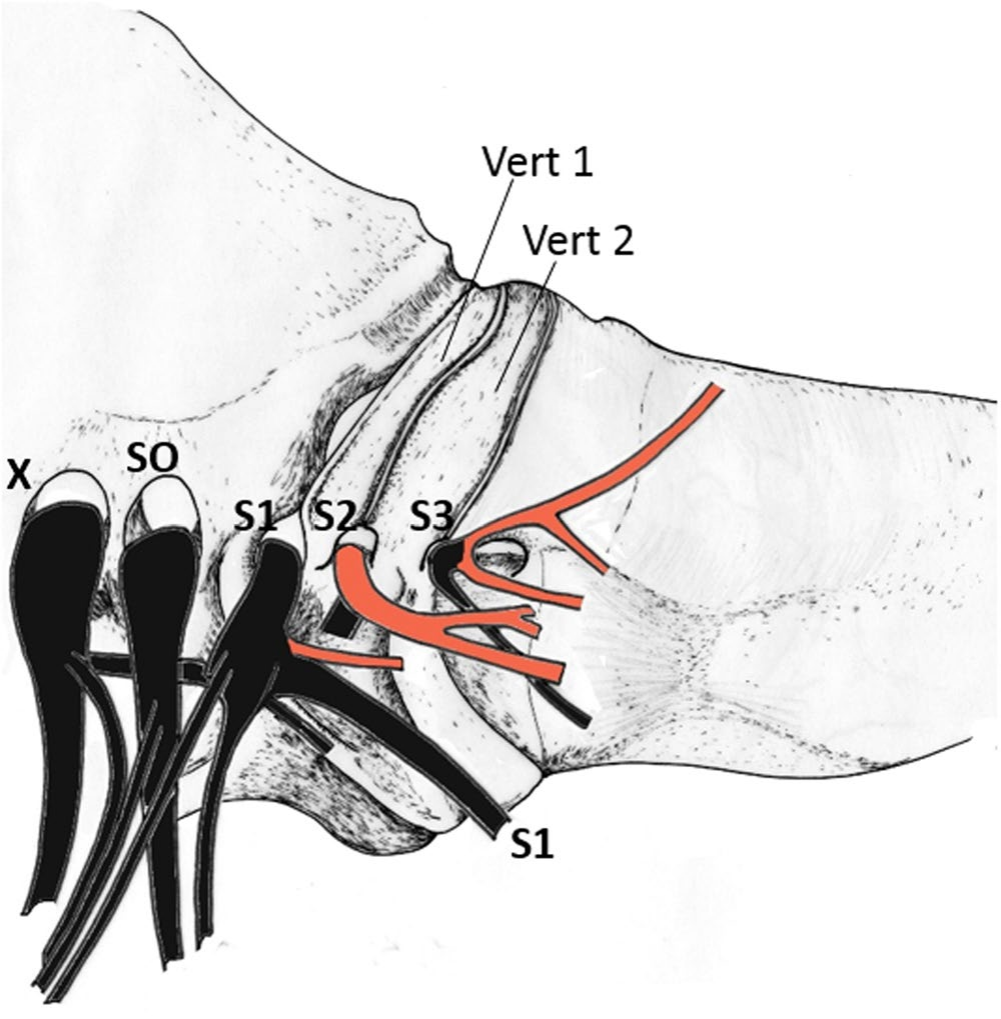
Left lateral view showing innervation pattern to the sonic muscles. Sonic branches are in red. SO, spino-occipital nerve; S1–S3, axon bundles of first to third spinal nerve; Vert, vertebral body; X, vagus nerve. Drawings were made by E.P.

The spinal cord in the Ostraciidae ends abruptly forming a cord-like filum terminale within the first vertebra^33^. Both sound-producing muscles were innervated by different branches originating from the myelencephalon, close to the caudal cerebellum, and the spinal cord (Fig. 4). Three nerve roots innervate the muscles. Ventral branches originating from the true spinal nerve one (S1) innervates sonic muscles. They are also innervated by spinal nerves two (S2) and three (S3). S1 and S2 innervate the extrinsic sonic muscles whereas S3 innervate both muscle.

Dissections reveal a similar morphology in *Ostracion cubicus*.

### Main sonic characteristics in *Ostracion meleagris*

**Figure 5:**
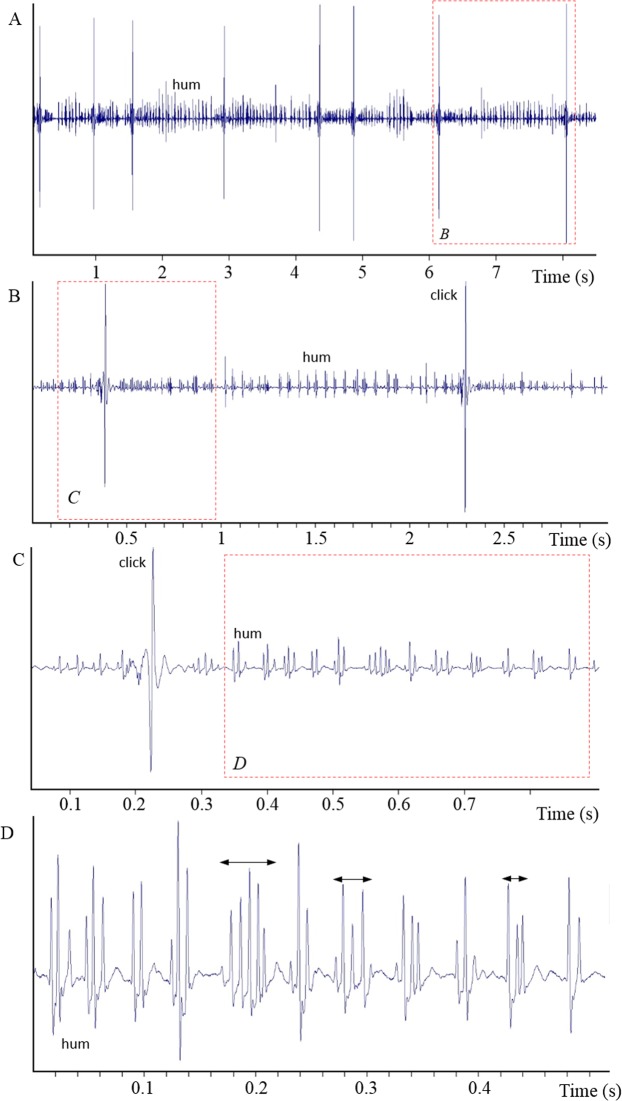
Oscillograms showing the hums and the clicks at different time scales in *Ostracion meleagris*. Red dotted rectangle refers to the next oscillograms. Double arrows correspond to the hum pulse duration. Note each pulse is made of different cycles that attenuate rapidly.

Two sound types (hum and click) were simultaneously recorded from the 27 hand-held fish (Fig. 5). Fish produced the hum continuously and regularly although they were sometimes interspersed with clicks. Hums were found in all recordings, but clicks were sometimes missing and were never produced alone.

**Figure 6:**
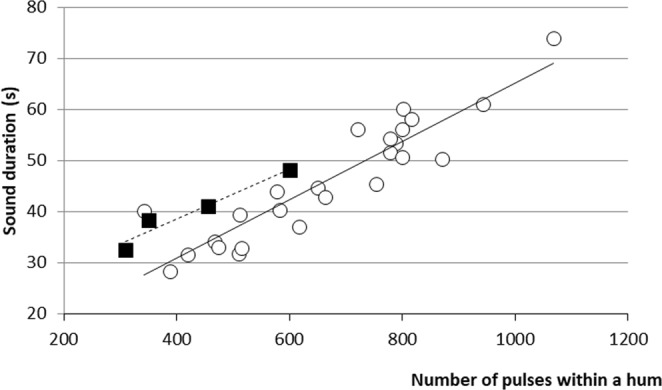
Relationship between the number of pulses and the call duration in the hums of *Ostracion meleagris* (white circles, y = 0.057x + 8.1323) and *Ostracion cubicus* (black squares, y = 0.0488x + 19.095).

**Figure 7:**
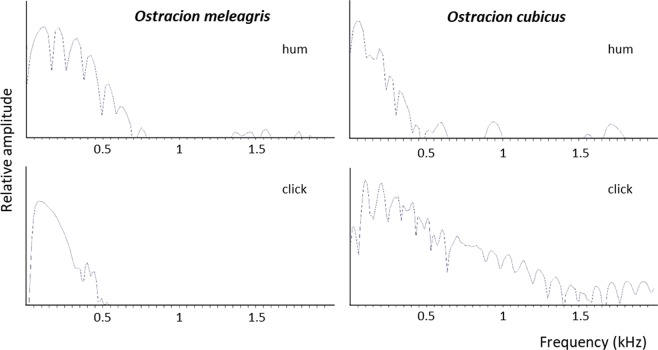
Power spectra of the pulses from hum and click in *Ostracion meleagris* and *Ostracion cubicus*.

Hums consist of long trains of 661 ± 274 low-amplitude pulses (mean ± s.d.; 27 specimens, 270 sounds, TL: 30–91 mm). The pulse period within trains averaged 65 ± 10 ms, and hum duration (mean 46 ± 17 sec) correlated with the mean number of pulses (r^2^ = 0.84; Fig. 6). The dominant frequency of hum pulses was 146 ± 5 Hz (Fig. 7).

Clicks consist of isolated pulses that were produced irregularly and randomly during the hums. The boxfish emitted from one to more than 50 clicks during the hum although click sounds are not found in all the hums. The time between two clicks varied from 58 ms to 41 s, meaning there is no repeatable click period. Pulse duration was 45 ± 4 ms (mean ± s.d.; 23 specimens, 230 sounds), and the fundamental frequency was 172 ± 5 Hz. Click amplitudes (1.7 ± 0.6 kPa, n = 170) had more than 10 times the sound pressure of hum pulses (0.14 ± 0.1 kPa, n = 170), equivalent to an amplitude difference of 20 dB.

**Figure 8:**
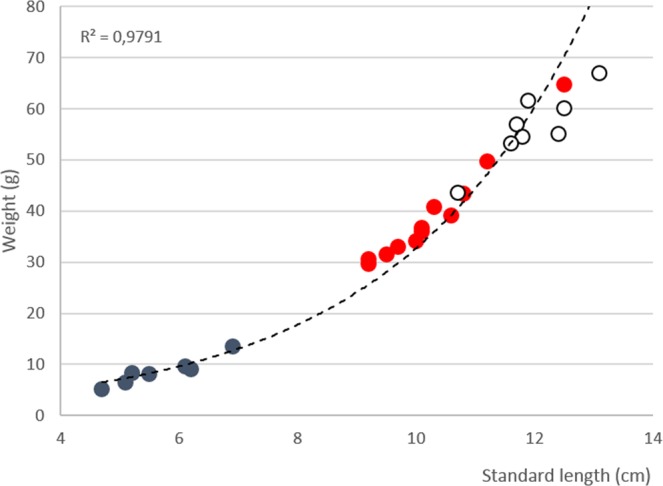
Relationship between body length and weight in *Ostracion meleagris*. Blue: juveniles, red: females; white: males.

### Intraspecific comparison of the calls in *Ostracion meleagris*

Fish weight correlated with standard length (r^2^ = 0.97, P < 0.001, n = 27) in juveniles, females and males (Fig. 8). Sound duration was 51 ± 17 s (n = 120) in males, 44 ± 18 s (n = 80) in females and 38 ± 10 s (n = 70) in juveniles (Table 1). However, no significant differences were found between the three groups for dominant frequency (KW, F_2,25_ = 2.22, P = 0.13), pulse period (F_2,25_ = 1.74, P = 0.19), sound duration (KW, F_2,25_ = 4.57, P = 0.1) and number of pulses (KW, F_2,25_ = 5.54, P = 0.06) for hums (Table 1).

**Table 1:** Length, weight and acoustic characteristics of sounds recorded in *Ostracion meleagris*.

	Males N_fish_ = 12 n_sounds_ = 120	Females N_fish_ = 8 n_sounds_ = 80	Juveniles N_fish_ = 7 n_sounds_ = 70
	$\bar{{\boldsymbol{x}}}\pm {\boldsymbol{s}}$	$\bar{{\boldsymbol{x}}}\pm {\boldsymbol{s}}$	$\bar{{\boldsymbol{x}}}\pm {\boldsymbol{s}}$
Standard length (cm)	7.9 ± 0.6	6.9 ± 0.8	3.8 ± 0.6
Weight (g)	51.5 ± 9.8	37.8 ± 11.5	8.6 ± 2.6
**Hum**	**N**_**fish**_ **= 12**	**N**_**fish**_ **= 8**	**N**_**fish**_ **= 7**
	**n**_**sounds**_ **= 120**	**n**_**sounds**_ **= 80**	**n**_**sounds**_ **= 70**
Call duration (s)	51 ± 17	44 ± 18	38 ± 10
Dominant frequency (Hz)	145 ± 5	148 ± 5	146 ± 4
Nbr of Pulses	749 ± 301	626 ± 254	523 ± 155
Pulse period (ms)	64 ± 9	66 ± 11	67 ± 10
**Click**	**N**_**fish**_ **= 10**	**N**_**fish**_ **= 8**	**N**_**fish**_ **= 6**
	**n**_**pulse**_ **= 100**	**n**_**pulse**_ **= 80**	**n**_**pulse**_ **= 60**
Dominant frequency (Hz)	174 ± 4	173 ± 7	170 ± 3
Pulse duration (ms)	45 ± 5	44 ± 3	43 ± 2

However, there was a positive relationship between the fish length and both the call duration (r_s_ = 0.61, p < 0.005) and number of pulses (r_s_ = 0.61, p < 0.005). Fundamental frequency (r_s_ = −0.23, p = 0.258) and pulse period (r_s_ = −0.21, p = 0.294) did not change with fish size.

The click dominant frequency was significantly different between the three groups (KW, F_2,950_ = 168, P < 0.05). Post-hoc comparisons indicated juveniles (169 ± 10 Hz, n = 320) were significantly different (Dunn’s test, P < 0.05) from both males (173 ± 10 Hz, n = 290) and females (172 ± 11 Hz, n = 340). Similarly pulse duration was significantly shorter (Dunn’s test, P < 0.05) in juveniles (43 ± 1 ms, n = 358) than in males (45 ± 0.5 ms, n = 825) and females (44 ± 0.2 ms, n = 655). However, both dominant frequency (r_s_ = 0.53, P < 0.001) and pulse duration (r_s_ = 0.29, p < 0,001) increased with fish size. Males produce more clicks per hum than juveniles (Table 1).

### Main sonic characteristics in *Ostracion cubicus*

**Figure 9:**
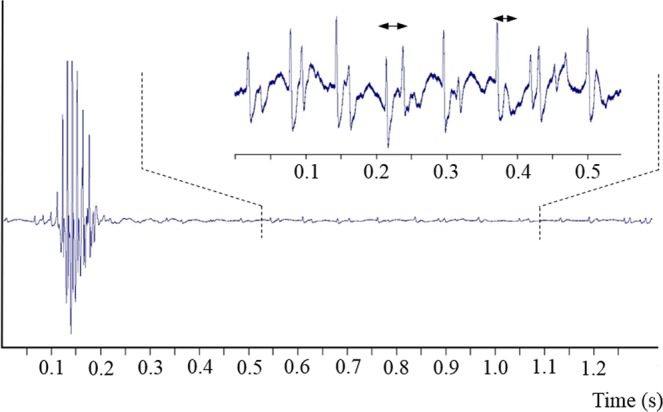
Oscillograms of a click and a hum (lower) and of a hum at an expanded time scale (between dotted lines) for *Ostracion cubicus*. Note the lower amplitude of the hum. Double arrows correspond to the pulse duration for a hum. Note the clicks differ from those in *O. meleagris*.

Again hums and clicks were recorded within a sequence (Fig. 9). Clicks (12.3 ± 2.6 kPa, n = 247) had 40 times greater sound pressure (32 dB increase) than hums (0.1 ± 0.05 kPa, n = 430). Hums lasted 40 ± 8 s and were composed of 425 ± 142 pulses with a fundamental frequency of 152 ± 4 Hz. Sound duration was related to the number of pulses (r^2^ = 0.94). Clicks in *Ostracion cubicus* differ from *Ostracion meleagris*. They consist of short trains of 5 ± 1 pulses lasting 56 ± 8 ms, with a dominant frequency of 189 ± 4 Hz. Clicks in *O. cubicus* provided a more complex power spectrum than in *O. meleagris* (Fig. 7).

The comparison of acoustic characteristics between *O. meleagris* and *O. cubicus* showed hums and clicks features were all significantly different (t-test, p < 0.05).

## Discussion

### Sound producing mechanism

Different fish species can use a common mechanism to produce different sounds^34–36^. In most cases, differences result from temporal variation dictated by the firing pattern of vocal pacemaker neurons^37,38^. In mechanisms that involve the swimbladder, sound production is generally determined by contraction of a single pair of muscles^2,18,19^. However, in some species within the Ophidiidae or Carapidae for example, two or three pairs of muscles can be involved^39–43^. The combined action of these different muscles can produce different sounds types^35,41,44,45^. However, the ability to produce two distinct sounds simultaneously with two crossing muscles appears to be unique. The sonic muscles of swimbladder-associated sonic mechanisms are classically innervated by the occipital nerve or spinal nerves^46–48^. There is usually not a combination of spinal and occipital nerves, most probably because of the ontogenetic origin of the sonic muscles^49^. They can be derived from muscles associated with the cranium or from lateral trunk (body) muscles^48,49^ as in both boxfish species. It is worth mentioning several families of catfishes include species that can also use different kinds of mechanisms that produce markedly different sounds. It is however easier to associate sounds and mechanisms in this taxa because these catfish use the pectoral spine to produce stridulatory sounds^50^ and swimbladder to produce drumming sounds^19,51,52^. In different Scorpaeniformes species (*Apistus* and *Pterois*), intrinsic and extrinsic swimbladder muscles have been described^53^ but sounds are still to be recorded. Also extrinsic muscles originate on the skull and not on the vertebral column as in boxfish. The complexity of the boxfish supports an important investment in this communication channel and suggests sounds should have important functions in the biology of this taxa.

**Figure 10:**
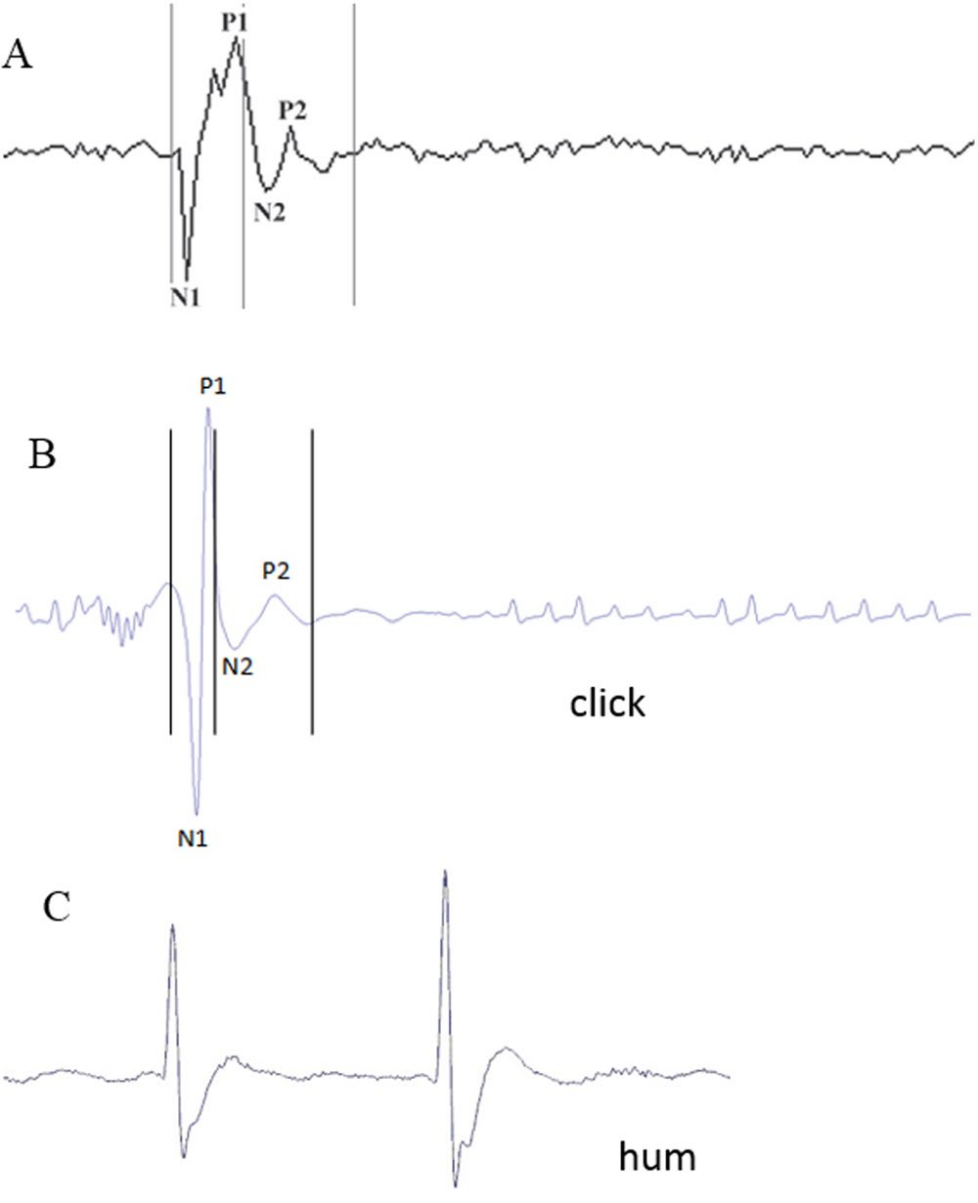
Waveform (**A**) of a sound induced by hammer strike in a male toadfish (Fine *et al*.^57^) and the waveform (**B**) of a click in *Ostracion meleagris*. Note the similarity between both sounds despite the swimbladder was excited in a different way. Displacement occurred over two cycles: an initial compression of the swimbladder (N1) followed by an expansion (P1) and a greatly attenuated second cycle (N2–P2) exhibiting rapid damping. The shape is inverted for the hum (**C**) where there is first a positive peak.

Sounds can be produced by superfast-contracting muscles^2,21,54^ whose contraction cycle provokes inward and outward movements of the swimbladder wall. This cause and effect relationships has been shown in the piranha *Pygocentrus nattereri*^55^ and the toadfish *Opsanus tau*^21,56^. In the study of acoustics of the swimbladder, the action of sonic muscles was mimicked by striking the swimbladder with a piezoelectric impact hammer^57^. These two movements, inward and outward, produced a negative and positive acoustic waveform, respectively. In *O. meleagris*, the oscillogram shape of the click sound is similar to the waveform resulting from the hammer strike in the toadfish (Fig. 10): a single rapid muscle twitch should create the click. In *O. cubicus*, the click call appears to be made of a train of successive cycles, indicating successive muscle contractions.

In the hums made by *O. meleagris* and *O. cubicus*, the pulse period corresponds to 65 ms (or 15 Hz) and 83 ms (or 12 Hz) respectively and not the dominant frequencies of 145 and 154 Hz respectively. Therefore the pulse period does not correspond to the muscle contraction rate. However, a deeper examination of hum pulses in the oscillogram reveals they can be made of 1 to 5 cycles having a period between 5 (200 Hz) and 8.5 ms (117 Hz) (Fig. 5D), which is close to the dominant frequency values. Therefore each pulse within the hums could result from regular packs of one to five muscle contractions (Fig. 5D). The waveform of the hum differs however from that of the click. Different hypotheses can be formulated. First, contraction amplitude is likely low in the hum causing shorter displacements of the swimbladder and weaker sounds. Second, the fast successive muscle contractions mask the end of individual hum cycles.

In the framework of this study, two different muscles work sequentially to produce a specific distress call consisting of hums and clicks. The muscles that produce the hum works for a long period of time (up to 130 seconds in this study!) and would be fatigue resistant. The click muscles have less sustained but more powerful contractions resulting in a greater amplitude sound than in the hums. Histological sections of fast sound-producing muscles show a common design: mitochondria are abundant in a large band of sarcoplasm in the fiber periphery, and a central core of sarcoplasm can be found in some but not all cases^51,58–60^. In *O. meleagris*, sonic muscles do not follow this scheme completely since large space devoted to sarcoplasm was not observed in all fibers. They are however characterized by an expanded development of sarcoplasmic reticulum and a low density of myofibrils that are related to fast contraction since the relative percentage by volume of myofibrils is inversely proportional to the speed and directly proportional to force^61^. The high concentration of sarcoplasmic reticulum is related to the ability to release and remove rapidly Ca^2+^ from the contraction site^62^. Ultrastructure and sound waveforms therefore support that both muscle pairs contract rapidly since dominant frequency is in the same order of magnitude in hums and clicks (146 *vs* 172 Hz).

Because we were unable to highlight qualitative differences between both muscles, we cannot support the association between a given muscle and a corresponding sound. We however have to suggest an alternate hypothesis. There is an overlapping innervation of intrinsic and extrinsic muscles meaning a click could result from the simultaneous contractions of both sonic muscles. The combined contraction would drive larger swimbladder movements and provoke louder sounds than each muscle separately. In this case, trains of contraction would produce the hum and the additional contraction of the second muscle would provoke the click. Physiological experiments are necessary to test our hypothesis.

### Acoustic signals

Of the three previously described sounds in *O. meleagris*^16^, only the spawning sound shares similarity with the hum because both are of long duration (>1000 ms). However, Lobel (1996) did not describe two sound types produced within a sequence. This difference may relate to the behavioral context in which sounds were recorded. Sounds in the previous study were recorded in the field during the reproduction period^16^. In the present study, fish were held by hand and recorded in a tank. In this situation, the behavior is usually associated with distress^63,64^. The two studies suggest a large sonic repertoire and supports the importance of acoustic communication in the Ostraciidae.

Two sound types were recorded within the same sequences: hums occur with sporadic clicks. These clicks are short (45 ms), have a dominant frequency of 172 Hz and are produced irregularly with a pulse period between 58 ms and 41 s. Clicks were thus irregular and would appear to result from isolated contractions rather than a repeated motor pattern. Hums however are long (*ca* 46 s), can possess hundreds of pulses and are regularly produced with a period between 40 and 90 ms. Therefore hums consist of trains of pulses produced by rhythmic neuronal firing^38,49,65^. Additionally, clicks have at least 10 fold greater amplitude (linear units) than hums in both species.

### Intraspecific differences

The hypothesized sound production mechanism in *Ostracion* species is supported by the intraspecific sonic characteristics in *O. meleagris*. In teleosts, sonic characteristic can vary with fish sex and size^66,67^. The amplitude variation is mainly related to the sound-producing mechanism. In species in which the muscle contraction rate determines the fundamental frequency, variations between specimen size or sex has little effect on timing of sonic muscle contraction and hence sound characteristic since it depends mainly on muscle twitch parameters^18^. In other words, in fish using fast muscles, the size can have a mathematical influence on the sound characteristics but the fish would not be able to distinguish such minor variation in frequencies^29,68,69^. In *O. meleagris*, fish size correlates with weight and separates into three classes: male, female and juvenile. Males (SL = 7.9 cm) are bigger than females (SL = 6.9 cm), both being obviously bigger than juveniles (SL = 3.8 cm). However, this size difference is not related to the dominant frequency, which differentiates only from 1 to 4 Hz (Table 1). In comparison, the difference would be about 400 Hz in pomacentrids with the same size variation^67^. Therefore dominant frequency does not provide information about the size or the sex in *O. meleagris*. The small differences between fishes having different sizes would be related to a scaling effect: longer muscles from larger fish takes longer to complete a twitch, providing lower frequencies^18,68^. In the same way, pulse duration does not provide intraspecific information. Similarly dominant frequency and pulse period of hum pulses did not differ between males, females and juveniles. If the expected status of distress call use to startle a predator or warn conspecifics can be confirmed, high intraspecific information content is not needed here. However, the sound duration indicated intraspecific differences since male sounds were longest (51 s), followed by females (44 s) and then juveniles (38 s).
